# Exploring Spatiotemporal Trends in Commercial Fishing Effort of an Abalone Fishing Zone: A GIS-Based Hotspot Model

**DOI:** 10.1371/journal.pone.0122995

**Published:** 2015-05-20

**Authors:** M. Ali Jalali, Daniel Ierodiaconou, Harry Gorfine, Jacquomo Monk, Alex Rattray

**Affiliations:** 1 Deakin University, Centre for Integrative Ecology, School of Life and Environmental Sciences, Faculty of Science, Engineering and Built Environment, Warrnambool, Victoria, Australia; 2 Department of Environment and Primary Industries, DEPI Queenscliff Centre, Queenscliff, Victoria, Australia; 3 Institute for Marine and Antarctic Studies, University of Tasmania, Private Bag 49, Hobart, Tasmania, Australia; 4 Dipartimento di Biologia, Università di Pisa, Pisa, Italy; California Polytechnic State University, UNITED STATES

## Abstract

Assessing patterns of fisheries activity at a scale related to resource exploitation has received particular attention in recent times. However, acquiring data about the distribution and spatiotemporal allocation of catch and fishing effort in small scale benthic fisheries remains challenging. Here, we used GIS-based spatio-statistical models to investigate the footprint of commercial diving events on blacklip abalone (*Haliotis rubra*) stocks along the south-west coast of Victoria, Australia from 2008 to 2011. Using abalone catch data matched with GPS location we found catch per unit of fishing effort (CPUE) was not uniformly spatially and temporally distributed across the study area. Spatial autocorrelation and hotspot analysis revealed significant spatiotemporal clusters of CPUE (with distance thresholds of 100’s of meters) among years, indicating the presence of CPUE hotspots focused on specific reefs. Cumulative hotspot maps indicated that certain reef complexes were consistently targeted across years but with varying intensity, however often a relatively small proportion of the full reef extent was targeted. Integrating CPUE with remotely-sensed light detection and ranging (LiDAR) derived bathymetry data using generalized additive mixed model corroborated that fishing pressure primarily coincided with shallow, rugose and complex components of reef structures. This study demonstrates that a geospatial approach is efficient in detecting patterns and trends in commercial fishing effort and its association with seafloor characteristics.

## Introduction

Globally, fisheries provide a pivotal source of food and income; hence, the sustainable maintenance of these limited renewable resources is critical to their longevity. However, detailed information about the spatial and temporal footprint of fisheries (i.e. the intensity and spatiotemporal variability) is often lacking [1–3]. In many fisheries, vessel monitoring systems are used to assess fishing activity and to inform marine spatial planning, particularly at large spatial scales [4, 5]. Compiling data from these sources has some constraints such as low spatial resolution, incomplete coverage of vessel tracks, and a lack of explicit linkages between data about actual fishing sites and CPUE reports [6, 7]. Recent assessments have shown that the benefits outweigh the costs of having access to detailed field-based information for both assessment and compliance purposes [5], leading to installation of global positioning system (GPS) units to geo-locate catch data on fishing vessels from small scale fisheries. GPS integration with data loggers have recently been trialed to assess fishing process in artisanal sea urchin [8] and scallop fisheries [9]. Due to the links between the spatial dimension of the distribution of a target species and the behavior of the fleet, spatially-explicit CPUE data increases the potential to decipher such connections [8, 10–12].

Marine resources do not exhibit random patterns, particularly in the case of benthic species, with stocks and species forming clusters (such as mussel beds) over local and regional scales [4, 13, 14]. Heterogeneity in species distribution may also result from species behavioral traits, population dynamics, habitat preferences, or fishing strategies [15]. In this regard, previous studies have trialed geo-statistical approaches, such as spatial autocorrelation and cluster analysis in assessing temporal (i.e. seasonal) and spatial patterns of exploitation among target species [16–18]. Examples of such research include predictive modeling of species abundance [19, 20], determining locations of biological hotspots and productive areas [21, 22], and monitoring fishing fleet activity to identify patterns in fishing pressure [23, 24]. The availability of GPS-enabled data loggers and their integration with geo-statistical approaches has the potential to provide new avenues for investigating patterns in productivity at hotspots identified across important fishing zones. In turn, this knowledge is expected to help with analyzing cumulative CPUE and spatiotemporal changes through time [10].

Identification of patterns of catch and effort in benthic fisheries is, however, not enough on its own to inform harvest strategies aimed at ensuring long term profitability. It is also important to understand how these patterns are associated with seafloor physical characteristics, because of a strong association between substrate structure and benthic species preferred habitat, upon which fishing pressure is superimposed [25, 26]. Acquiring this type of data has been limited in the past. Newly established remote sensing instruments, such as LiDAR systems might provide an opportunity to identify seafloor characteristics of exploited mollusk habitat. Bathymetric LiDAR uses laser pulses to acquire feature characteristics by recording the signals reflected from the seabed and the ocean surface to infer depth [27]. LiDAR-derived digital elevation models (DEM) have been applied to generate marine-based 3-D architecture in shallow marine habitats [28]. Seafloor features may be determined that influence both the habitat preferences of aquatic species and the scale and pattern of demersal fishing [23, 29].

Blacklip abalone (*Haliotis rubra*) is a commercially important mollusk in Australia. It is endemic to the region of southern Australia extending from mid New South Wales along the mainland east coast to as far as the south west of Western Australia as well as the coastal waters of the island state of Tasmania. This species represents a high proportion of Australia’s abalone wild fishery, comprising 82% of the total catch landed during 2010 [30]. In Victoria, however, harvest quotas have declined substantially in recent years, with several factors suggested, including illegal fishing, the creation of no-take marine parks, overfishing, and disease [30]. As a consequence, various strategies have been adopted to enhance management of the fishery [30, 31]. These have generally focused on increased spatial resolution in assessment and management coupled with greater stakeholder engagement in co-managing the fishery. In terms of assessment this has included the use of GPS data loggers to geo-locate catch data, structured or directed fishing approaches to harvest abalone from specific stocks recovering from disease impacts, and acquiring improved knowledge about the spatial trends in growth and maturation rates in south west fishery of Victoria [32]. It was in this context that this study focused on analyzing CPUE data for *H*. *rubra* using GIS-based spatial statistics. Specifically, we aimed to (1) determine the spatial and temporal trends in the distribution of CPUE, and identify productivity hotspots, and (2) assess associations between the patterns of CPUE and the structure of the seafloor at the reef scale by using high-resolution LiDAR-derived seafloor variables. The information presented here is anticipated to provide an insight into the key determinants of catch and effort patterns in benthic fisheries. The knowledge acquired may be applied towards better informed selection of harvest strategies that balance or optimise financial profits for fishing enterprises while obtaining improved outcomes in ecological performance such as preventing stocks to be overharvested.

## Materials and Methods

### Ethics statement

Fishing effort data for blacklip abalone was captured by the Western Abalone Divers Association (WADA) as part of annual commercial quota licence agreement for reef zones designated by the Department of Environment and Primary Industries for state managed waters of Victoria. The field studies did not involve endangered or protected species. GPS coordinates of the study area location can be found on each of the geographic maps.

### Study site

**Fig 1:**
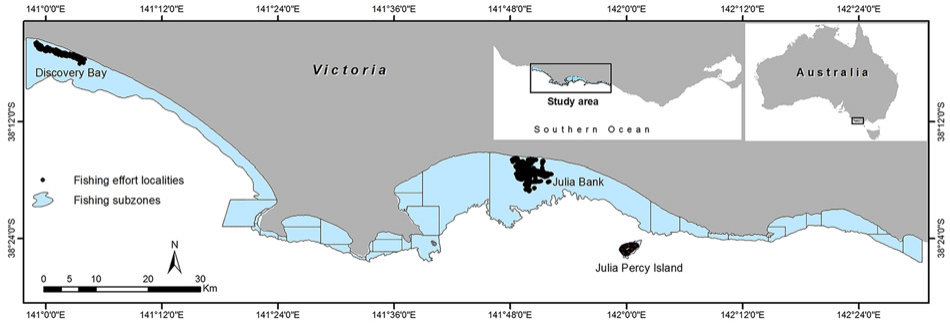
Map of the study area. Fishing subzones and fishing effort localities at Discovery Bay, Julia Bank, and Julia Percy Island subzones along the south west coast of Victoria, Australia.

**Fig 2:**
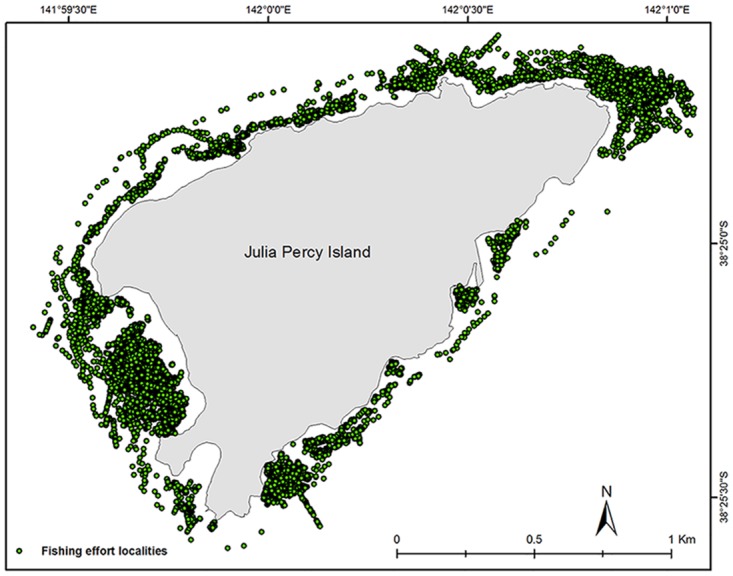
Distribution of GPS records around Julia Percy Island. An example of the distribution of fishing effort recorded by GPS units around Julia Percy Island.

The study area encompassed the western abalone fishing zone on the south west coast of Victoria, Australia. This is a statutory management region that extends from the Hopkins River in Warrnambool to the Victorian-South Australian interstate border on the western side of Discovery Bay (Fig 1). Geographically, the area was bounded by 140° 56' to 142° 31' E and 38° 06' to 38° 26' S, with a coastal length of approximately 200 km. The exposed open coast encompassed a mosaic of reef and bare sediment, with near shore patchy reef extensions from the intertidal zone to deeper offshore waters. Geomorphology across the study area ranged from low to high complexity. Algal assemblages were mostly dominated by kelp and fucoid beds that serve as appropriate habitats for many benthic fish and invertebrates. Three of the main abalone fishing subzones, Discovery Bay, Julia Bank and Lady Julia Percy Island (Fig 2), were selected for the assessment of CPUE distribution patterns. These subzones contain some major reef extensions of interest to commercial fisheries and despite their disaggregation they have in common that all were unaffected by a novel outbreak of the disease abalone viral ganglioneuritis (AVG) which decimated populations of abalone (*H*. *rubra*) in adjacent subzones during 2006–08 [33]. Apart from absence of disease, these three subzones were selected because of: (1) the availability of GPS records of commercial diving locations for abalone from 2008 to 2011, (2) the high numbers of abalone that have been harvested from these areas in recent years, (3) diversity in specific seafloor characteristics such as geology, rugosity and depth. These reefs became the mainstay of the fishery during the period immediately post-disease while other areas of the zone remained closed for several years in response to the AVG outbreak [34].

### Data acquisition and analysis grids

Catch and fishing effort data from six commercial divers harvesting *H*. *rubra* were provided by WADA for the three study subzones (total ~1422 diving hours). These data were recorded using a boat-based GPS system that logged catch localities and size information for years 2008–2011, a period during which much of the zone was closed to fishing to promote post-disease recovery. However, 8 subzones from the entire western zone were open to blacklip abalone fishing operations, of which, GPS records from 3 subzones were considered due to the quality and continuity of their records over the four-year study period. Commercial harvest of abalone is undertaken using a boat equipped with a hookah system that delivers compressed air from the surface to the diver underwater via an umbilical pressure hose of approximately 100 m in length. GPS units integrated into electronic shellfish measuring boards were mounted onboard individual fishing vessels. These systems log data about a diver’s catch during a single fishing event. As bags of abalone are brought aboard a diver’s vessel each abalone is passed (swiped) through the spring-loaded jaws of the measuring board which records the maximum shell length, time, date, latitude and longitude [10]. One potential issue with such a system is that the geo-located data logged is indicative of the swipe not the dive locality; we assumed that swiping occurred proximally to catch locations because divers usually stay within less than 100 m of the vessel to reduce umbilical drag [35]. The main violation of this assumption was in those instances where the vessel drifted away from the dive site before the catch was measured by swiping through the measuring machine. Outliers were removed from the data prior to analysis. These outliers were identified by querying time stamps to identify high vessel speeds indicative of transit between sites [10]. This was also corroborated using LiDAR identifying mismatch between reef locations and diver records. Fine-scale rectangular grid-based maps of 1 ha cell size (100 m × 100 m) were created for each study site using the Repeating Shapes ArcGIS extension tool [36]. The amount of catch (kg) for each abalone diver on each fishing day was estimated by using the allometric relationship: W = 0.000412(SL/10)^2.76^ where SL is swiped-abalone shell size in millimetres [37]. Reported catch was then divided by fishing hours derived from fishers’ logbook data to estimate CPUE. We assumed that effort was evenly distributed across GPS locations. CPUE estimates were then derived for each grid by a spatial join in ArcGIS 10 (ESRI) to collate all diver records (CPUE) for each 1 ha cell.

### LiDAR-derived seafloor topographic variables

**Fig 3:**
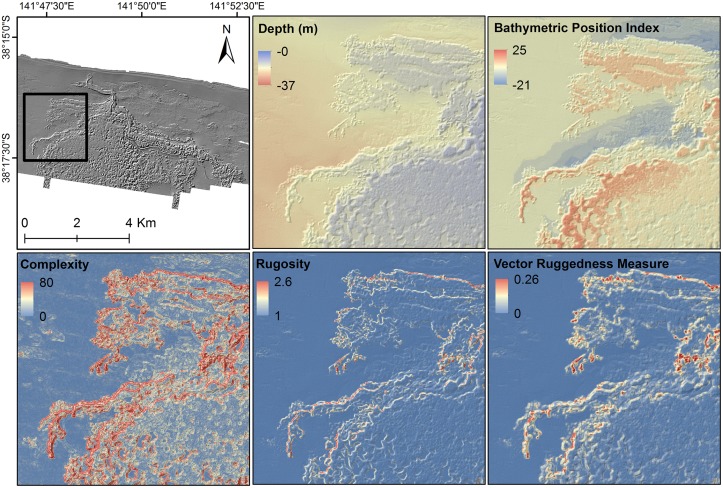
LiDAR-derived seafloor variables. Artificially illuminated LiDAR bathymetry of the study area magnified over the Julia Bank fishing subzone and examples of the five LiDAR derived seafloor topographic derivatives.

The airborne LiDAR bathymetric data used in this study were acquired through Fugro LADS Corporation Pty Ltd in 2007 for the entire coastline of Victoria, Australia. All LiDAR data were collected using a LADS Mk II acquisition system coupled with a GEC-Marconi FIN3110 internal motion sensing system and a dual frequency kinematic GPS. This system was mounted to a DeHavilland Dash-8 aircraft using a fixed wing platform. The flight lines were spaced at approximately 220 m, with an acquisition swath width of 240 m, leaving a swath overlap of around 10 m. The LiDAR system contains two laser scanners: (1) a near infrared laser at 1064 nm, which is reflected at the water surface, and is used to collect topographical data and (2) a green laser at 532 nm, which is continuous in the water column, and is used to capture the reflectance of the laser light from the seabed. Elapsed time between two echo pulses and the speed of light in the water determines the seabed depth. Primary point soundings and LiDAR bathymetry data were gridded in a 5-m DEM as a continuous representation of the seabed surface. This DEM had a maximum depth of 37 m, and was used to generate a suite of secondary products referred to as seafloor topographic derivatives (Table 1). These variables were selected for several reasons: (1) their potential ability to capture variation in seafloor roughness, (2) their probable importance in determining the distribution of abalone assemblages, and (3) their likely impacts on the strategies of fishing operations. These derivatives represent variation in seafloor characteristics, and susceptibility to sediment accumulation (bathymetric position index [BPI]) and the surface area of the reef structure (complexity, rugosity and vector terrain ruggedness [VRM]) (Fig 3). Collinearity between derivatives was checked to retain the least correlated variables in the analyses. Therefore, complexity and BPI were kept and other variables including rugosity and VRM were eliminated due to high correlation with complexity (>0.7). The BPI value provides an indication of whether any particular pixel forms part of a positive (e.g., crest) or negative (e.g., trough) feature of the surrounding terrain. The BPI is based on the variation among cells within a specified radius or annulus; it may be calculated at a variety of user-defined scales so as to capture local and broad-scale variations in bathymetric position. In this study, BPI was calculated at an outer radius of 150 m and an inner radius of 50 m. The rugosity is seabed roughness with lower values indicating smooth areas and higher values showing high-relief regions. VRM shows variations in terrain ruggedness with values from 0 (no terrain variation) to 1 (complete terrain variation). Topographic complexity is a second derivative of the slope surface. This factor has also been described as a major driver of ecosystem structure and function that can influence multitude of process including species abundance [25].

**Table 1:** Secondary derivative products generated from LiDAR bathymetry.

Layer	Variable definition	Software
Bathymetry	Provides a measure of depth for the entire study area.	ENVI 4.7
Bathymetric Position Index	BPI is a measure of a defined elevation at a special location relative to the overall landscape, and involves the difference of height at a focal point compared to the mean elevation of surrounding cells. Locations with higher elevation have positive values, while lower elevations have negative values. Values near zero represent flat regions [38].	ArcGIS extension benthic terrain modeler (BTM version 1.0) [39]
Complexity	Second derivative of the slope surface indicating the rate of change in slope values. This parameter encompasses the three-dimensional arrangement of structural elements over a seafloor surface [29].	ENVI 4.7
Rugosity	The rugosity is topographic roughness with the ratio of the surface area to planar area across the neighbourhood of the central pixel.	ArcGIS extension benthic terrain modeler (BTM version 1.0)
Vector Ruggedness Measure	VRM is terrain ruggedness indicating the variation in three-dimensional orientation of grid cells within a neighbourhood.	ArcGIS extension benthic terrain modeler (BTM version 1.0)

### Spatial autocorrelation and hotspot analysis

Spatial statistics tools in ArcGIS 10 Software (ESRI) were used to analyze spatial and temporal patterns in CPUE data. Global Moran's I [40] was applied to compute autocorrelation in CPUE within the 1 ha analysis grids for each year. Using the distance, location, and values of cells, Moran’s Index was calculated with values ranging between -1 (dispersed pattern) and +1 (clustered pattern), with values near zero indicating random distribution. Several distance classes (including 125, 250, 500, 750, 1000, and 1500 m) were considered to determine the distance band where autocorrelation and clustering patterns occur in CPUE distribution. This approach evaluates whether CPUE across the space of analysis grids occur non-randomly and if so, then whether these are dispersed or clustered. Fixed distance band and Euclidian distance were adjusted for the autocorrelation analyses.

Once the global patterns in the dataset were determined, the local Getis-Ord Gi statistic [41] was used to determine those areas with high and low values of CPUE, which were designated as hotspot and coldspot areas, respectively. This approach determines statistically significant local autocorrelation and dependence among neighboring cells. The 250-m distance band was chosen for hotspot analysis following the analysis of Moran’s autocorrelation where this distance band resulted in high z-score values as an indication of clustering patterns in CPUE data. This threshold selection was also on the basis of the scale of the analysis grids.

Significant values of hotspots analyses (z-score > 1.65) were extracted from each hotspot map to make a binary layer for each year. Output hotspot maps were then classified into 4 classes according to the significant values of z-score using the union overlay tool in the ArcGIS environment to generate four-year cumulative hotspot maps. Each hotspot class illustrates the particular area based on number of years fished, with classes 1 to 4 indicating whether a specific region was characterized by one to four years with significant hotpots indicative of sustained fishing pressure.

### Integration of CPUE with LiDAR derivatives

Mean values of LiDAR derivatives within each grid cell across two study subzones (Julia Bank and Discovery Bay; where bathymetric information were available) were extracted to integrate with four-year log-transformed CPUE data. Within this framework, generalized additive mixed model (GAMM) with a Gaussian distribution was used to model the relationship between CPUE and LiDAR-derived complexity, depth and BPI data. This modeling approach is a non-parametric regression method that is capable to account for dependence between observations by adding a correlation structure to the additive model. GAMM was constructed using the gamm function within the mgcv R package (R 2.15.3) [42]. Covariates were fitted as smooth functions using a thin plate regression spline and year was used as a random effect. Model selection was based on the lowest akaike information criterion and a visual examination of residual plots. The boundaries of targeted reefs were also digitized to identify reef areas using available LiDAR coverage. Reef area was then compared against CPUE patterns to quantify the proportion of the reef extent that was fished over four years within the study subzones.

## Results

### Spatial autocorrelation

**Fig 4:**
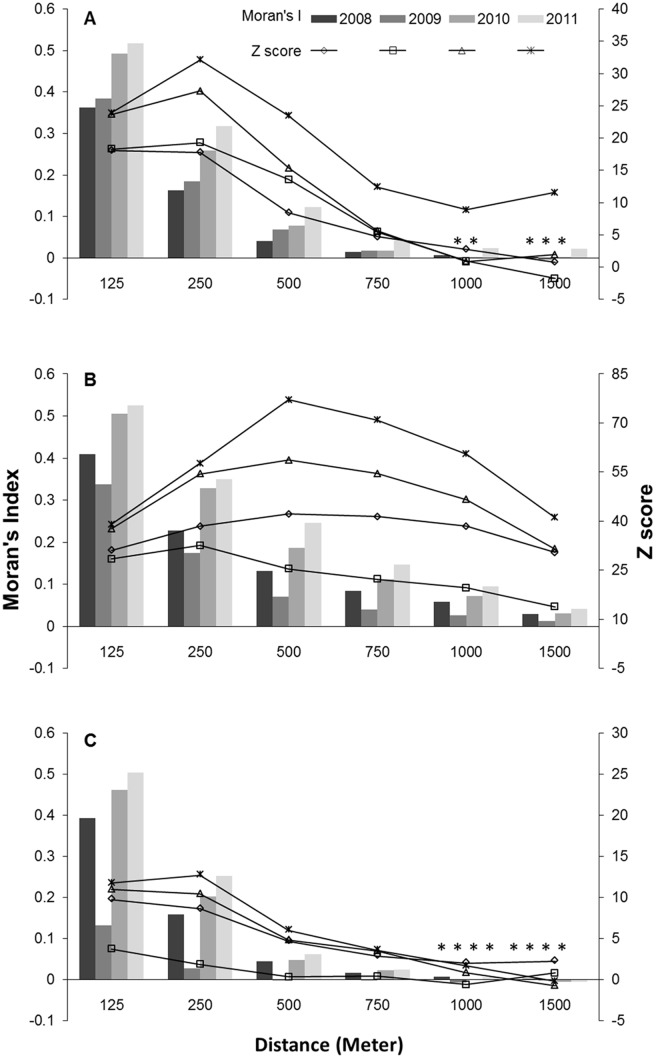
Moran’s Index and Z-scores in the variety of distance bands calculated for four study years. Moran’s Index and Z-score calculated for each year at Discovery Bay (A), Julia Bank (B), and Julia Percy Island (C) analysis grids. Values greater than zero indicate the clustering of fishing effort (positive spatial autocorrelation), whereas values less than zero indicate dispersed fishing effort (negative spatial autocorrelation). Non-significant distances (p-value >0.05) are shown with star (*) for Moran’s I values.

Across the three fishing sites, Moran’s I analysis showed that CPUE was spatially clustered. Most significant clusters were found at shorter distances. For instance, high clustering was often observed at 125, 250, and 500 m distance bands, with Moran’s I decreasing at larger distance thresholds. Moran’s I and z-score values were higher in 2010 and 2011 compared to 2008 and 2009 for the three analyzed subzones (Fig 4). In addition, higher Moran’s z-score values were obtained at Julia Bank compared to the other two study subzones (Fig 4B).

### Hotspot analysis

**Fig 5:**
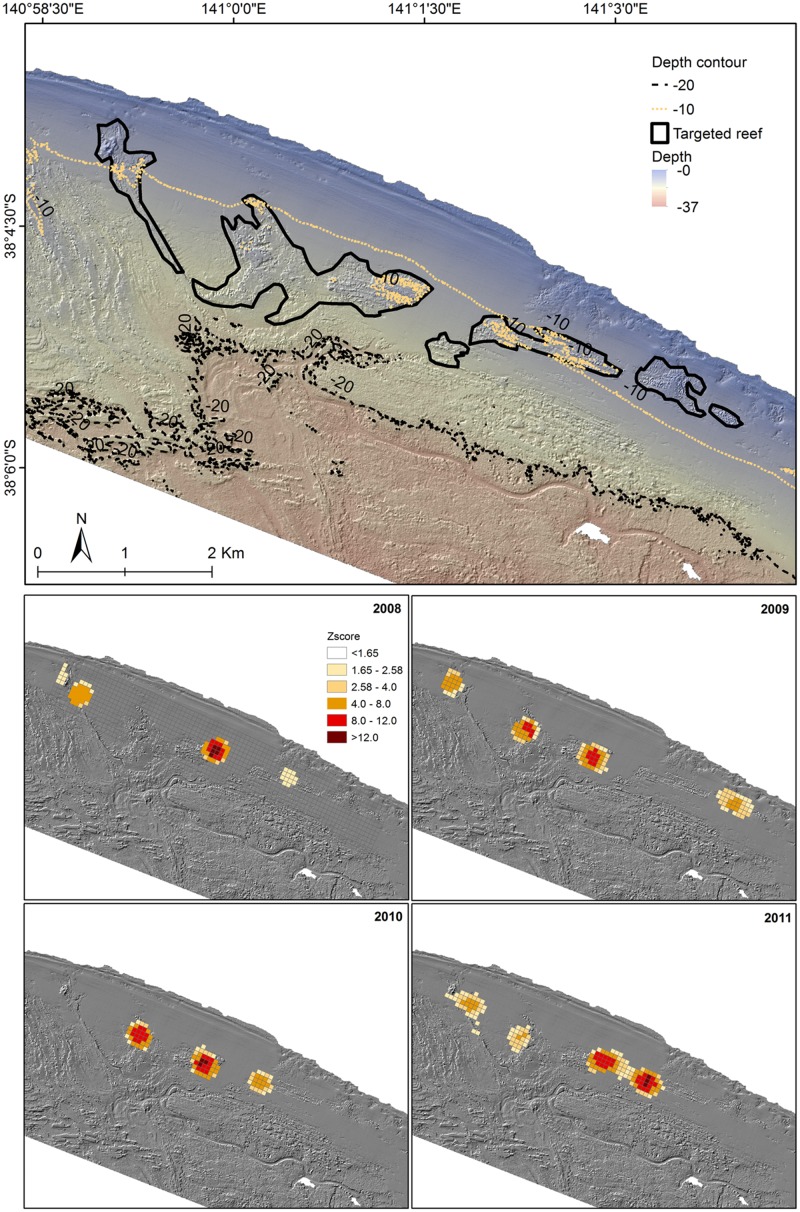
Hotspot analysis of CPUE distribution at Discovery Bay. Hotspot analysis of CPUE distribution from 2008 to 2011 at Discovery Bay over LiDAR derived hillshade showing the boundaries of the targeted reefs on the top panel.

**Fig 6:**
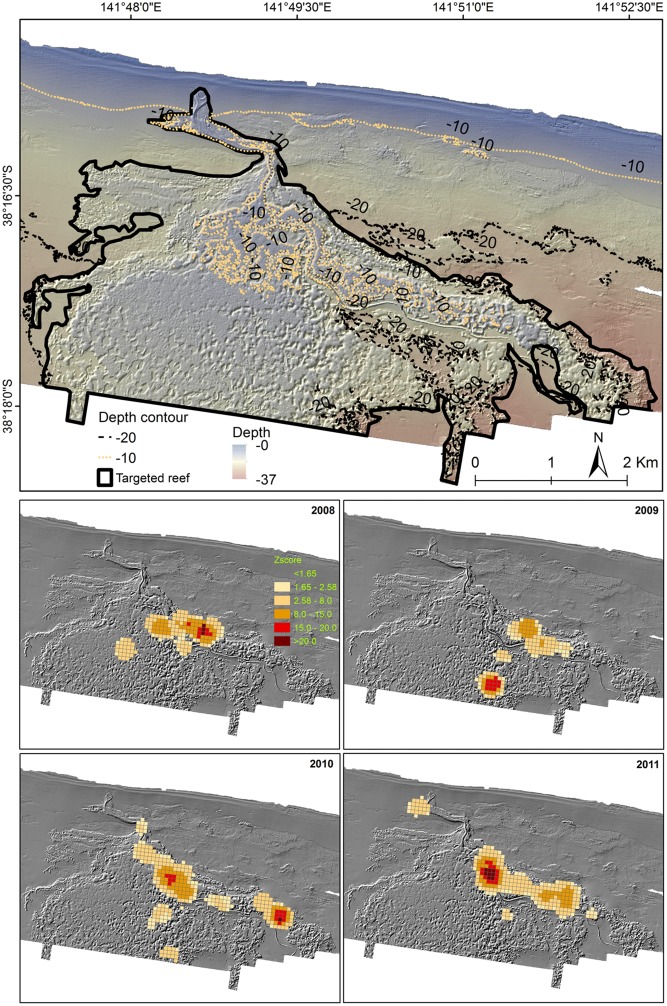
Hotspot analysis of CPUE distribution at Julia Bank. Hotspot analysis of CPUE distribution from 2008 to 2011 at Julia Bank over LiDAR derived hillshade showing the boundaries of the targeted reefs on the top panel.

**Fig 7:**
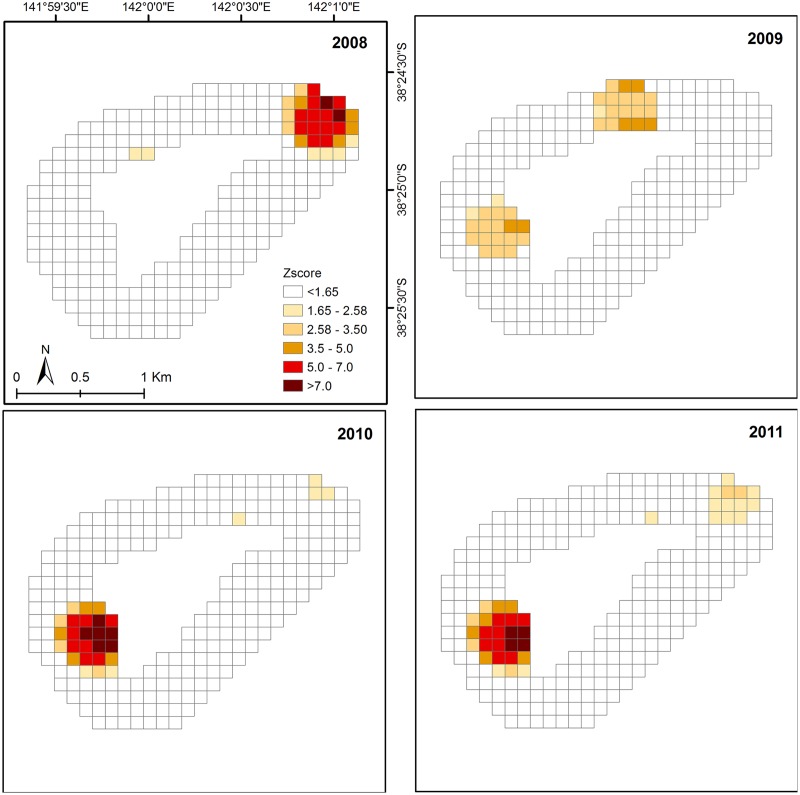
Hotspot analysis of CPUE distribution at Julia Percy Island. Hotspot analysis of CPUE distribution from 2008 to 2011 at Julia Percy Island (LiDAR data were not available).

**Fig 8:**
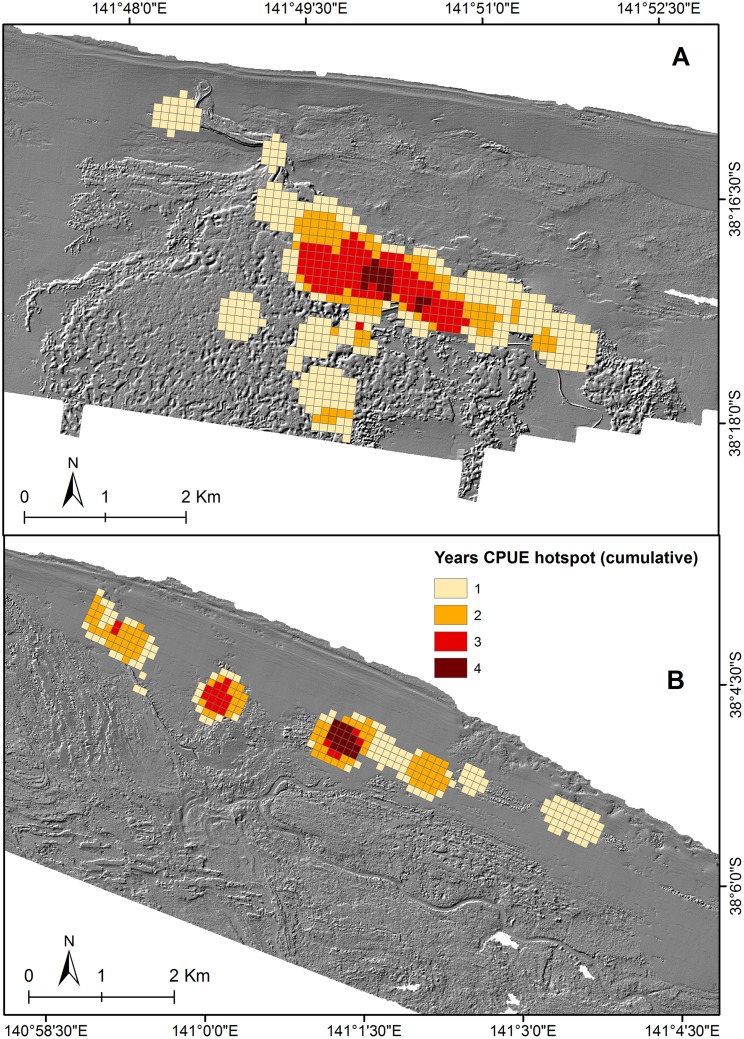
Cumulative hotspot distribution map. Cumulative CPUE hotspot map overlays (based on the number of years that CPUE was clustered) for (A) Julia Bank and (B) Discovery Bay over LiDAR derived hillshade.

The results of the local Getis-Ord Gi statistic indicated that the observed clustering patterns in CPUE were caused by the accumulation of grid cells with high values of fishing effort intensity in particular regions (Figs 5–7). Significant spatial and temporal shifts in CPUE between 2010 and 2011 were observed. In addition to the observed trends, the intensity and size of hotspots at Julia Bank tended to be larger nearer to the center of this subzone (Fig 6). In comparison, these patches often occurred in the southwest and northeast of the region at Julia Percy Island (Fig 7). The overlaying of hotspot data layers classified as temporal coverage classes showed spatial variation in CPUE over the four years. Hence, the persistence of CPUE hotspots indicated temporal consistency in the spatial location of hotspot regions (Fig 8). The results clearly showed that commercial divers targeted certain sites across the four study years, with hotspot regions being temporally concentrated in the center of both Julia Bank and Discovery Bay, and to the south west of Julia Percy Island.

### Integration of CPUE and LiDAR bathymetry

**Fig 9:**
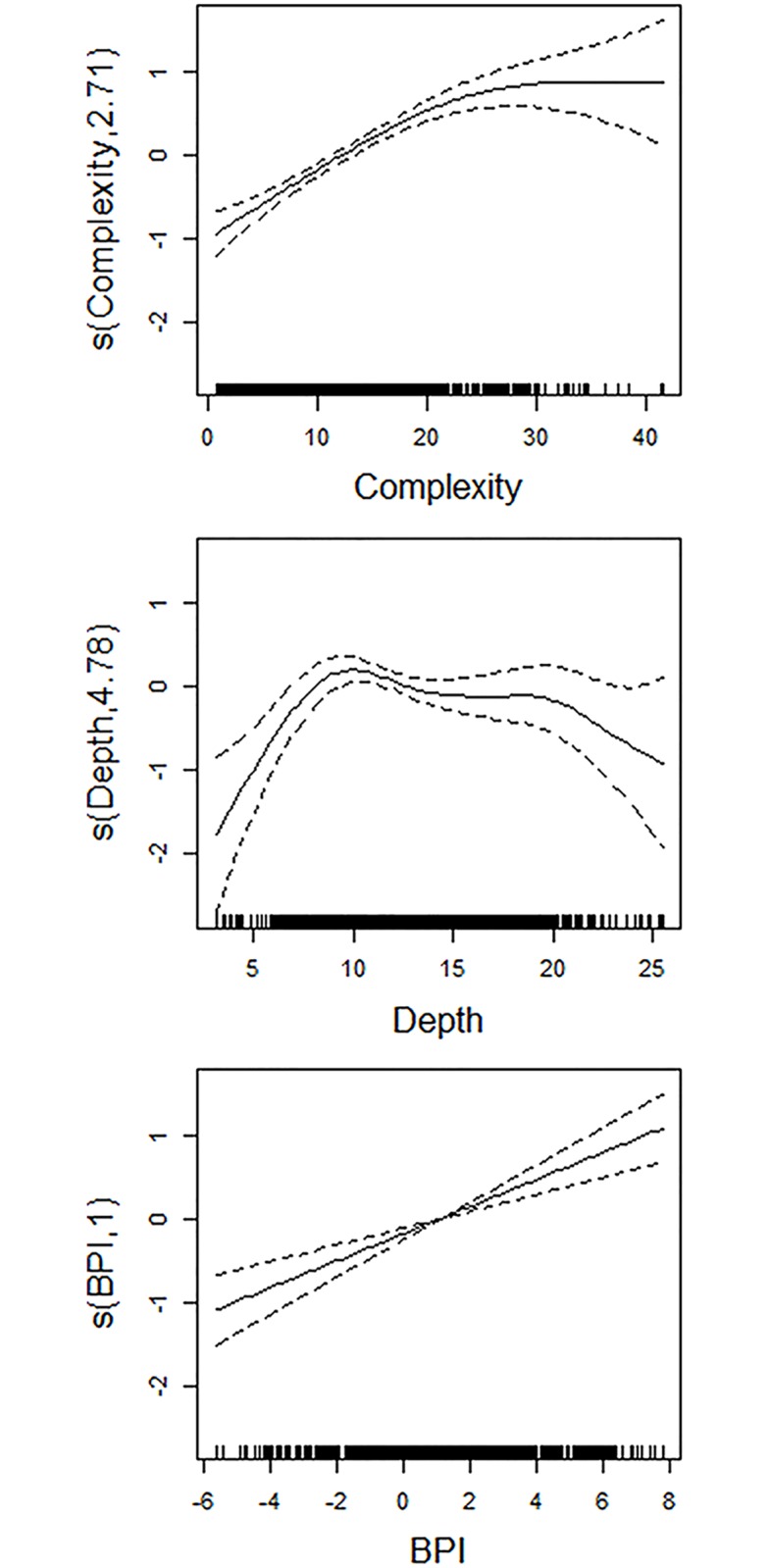
Model terms for the four-year generalized additive mixed model of blacklip abalone CPUE. Estimated smooth functions (solid lines) with 95% confidence interval (dashed lines) are shown for each explanatory variable. Y-axis = fitted function with estimated degrees of freedom in parenthesis; X-axis = variable range with rug plots indicating sampled values.

**Fig 10:**
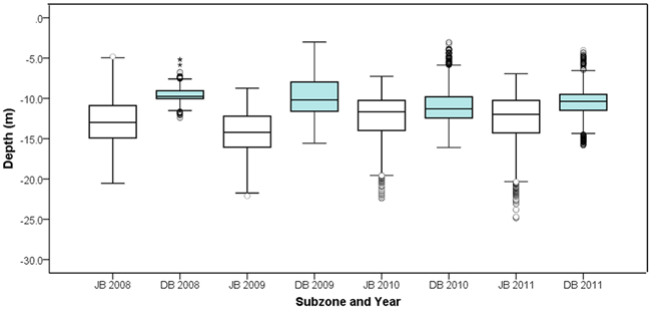
Bathymetric boxplot of GPS data loggers from blacklip abalone fishing activity. Bathymetric boxplot of GPS data loggers used to record information on blacklip abalone commercial fishing effort from 2008 to 2011 at two fishing subzones, Julia Bank (JB) and Discovery Bay (DB) along the south west coast of Victoria, Australia (number of divers = 6; total effort = 1422 hours).

The integration of four-year CPUE with seabed topographic variables using GAMM indicated a significant association with reef structure and depth (Table 2). According to GAMM results, CPUE distribution was associated with high reef complexities, and CPUE values increased with increase in seabed complexity (Fig 9). The relationship with BPI provided that CPUE rates were dense toward areas identified as crest (ridge) or trough (valley) (BPI values > 0 and values < 0 respectively) with comparably small CPUE rates falling on flat bottoms (BPI values = 0). Smooth curve for depth showed abalone CPUE from this study mostly tended to occur in shallow waters of about 10 m (Fig 9). Further exploration of bathymetric layers revealed the ranges of diving depths over fishing years; from 24.8–4.8 m at Julia Bank and 16.0–3.0 m at Discovery Bay (Fig 10). Interestingly, at Discovery Bay, none of the fishing effort localities occurred at depths greater than 16 m, with the reefs at this site being about 9 m shallower compared to Julia Bank. Comparison between CPUE patterns (area h) and reef area showed that ~ 30% of the fishable reefs extent at Julia Bank was targeted over four years while this was ~ 60% for the reefs at Discovery Bay. More exploration of CPUE patterns within the targeted reefs also indicated that ~20 (at Julia Bank) to 50% (at Discovery Bay) of the reefs with optimal diving depth 5-15m were under fishing operations over four years.

**Table 2:** The approximate significance levels (*p*-value), estimated degrees of freedom (edf) and F statistics (F stat.) for each of the explanatory variables used in generalized additive mixed model applied on blacklip abalone CPUE data.

Parameters	edf	F stat.	*p*-value
Complexity	2.71	34.8	<0.001
Depth	4.78	5.0	<0.001
BPI	1.0	27.7	<0.001

## Discussion

Geospatial approaches applied in this study demonstrated the spatiotemporal patterns and clusters in the distribution of CPUE for the commercially important blacklip abalone. Integrating bathymetric LiDAR data and CPUE using GAMM indicated that CPUE mainly coincided with complex reef structures in shallow waters. Until relatively recently, the resolution of marine environmental data has been too coarse to compare with resource exploitation. The availability of both bathymetric data and precisely positioned fishing effort localities enabled us to utilize geo-statistical approaches that better reflect the scale at which trends in effort target the resource.

Although analyses of CPUE patterns are frequently considered in fisheries, these patterns are often assessed at a coarse scale (kms) that is much larger than the scale of harvest [43]. In this study, GPS-based individual effort records provided an improved data source at finer spatial scale. A recent study trialing grid based analysis of GPS data for monitoring small-scale diving for the sea urchin (*Paracentrotus lividus*) also demonstrated the usefulness of fine-scale benthic fisheries assessments [8]. In common with many other fisheries, our study showed that abalone fishing is a non-random and heterogeneously distributed process [44, 45]. At an appropriate analytical scale, patterns in CPUE can be expected to provide evidence about whether shifts or continuity in fishing pressure is occurring in response to a given fishing strategy. It might also be potentially useful in providing trends in stock dynamics across particular zones. For example, in the case of spatially structured target species such as abalone, when the abundance of a stock begins to decline commercial divers tend to explore a more expansive area of reef in order to maintain catch rates [46]. Consequently, divers spend less time at sites with low aggregations of abalone, and subsequently move to alternative stocks in preference to persisting with reducing catch rates. This behavior reduces relative effort concentration, and CPUE become more evenly spread across a particular fishing zone. It may also create hyper-stability in CPUE making it a poor indicator of abundance or biomass until the resource becomes severely depleted. However, fishermen generally apply more effort to sites that have provided historically higher catch rates or return to known zones of high productivity [47].

In addition, the observed CPUE distributional patterns are likely caused by the preferences of individual divers with regard to environmental constraints or stock dynamics. Although the behavior of abalone fishers were poorly understood in the past [48], they often prefer fishing grounds close to ports and avoid adverse weather conditions and exposure to high wave energy [49]. Reducing travel time at sea and associated fuels costs are also important factors that influence the choice of fishing location. More accessible areas often receive high fishing pressure eventually making them unattractive to divers due to low yields caused by overexploitation. This preference for intensively fishing the more accessible locations makes abalone stocks vulnerable to serial depletion that often ultimately leads to stock collapse [50]. One possible solution to this problem suggested by Prince [51] is to match the scale of management to the scale of resource dynamics expressed as micro-management for micro-stocks. This contrasts with current practice where statutory management arrangements applied by Government agencies occur at a broad scale, typically 100s km, while abalone dispersion and fishing activity occur at the small spatial scale (10-100s of meters). However, using cost-effective and precise GPS tracking might provide managers an effective snapshot of spatial and temporal changes in CPUE under the current harvesting regime and at the individual reef scale. Such spatial assessments can also assist towards selecting appropriate fishing strategies such as implementing regulated catch caps and rotational fishing patterns at subzonal resolution to prevent stocks becoming overfished.

The GIS-based analyses used in this study also revealed the existence of clustering patterns and hotspots in abalone CPUE, with temporal trends being observed across short distance thresholds. In nature, organisms do not often exhibit uniform or random distribution, but aggregate in some type of spatial structure or patch [52]. In the context of fisheries, particularly benthic fisheries, the patchy spread of catch and effort is linked to the aggregation and occurrence of targeted species likely driven by suitable structure characteristics (i.e cryptic habitat availability) and oceanographic parameters (i.e exposure) [8, 53]. This phenomenon highlights the advantages of the spatial assessment of a fishery because it enables the location and intensity of fishing effort to be identified, along with how this information connects to target species productivity [18, 54]. For instance, CPUE hotspots that were observed to be consistent over time, as observed in the cumulative maps (Fig 8), might indicate the presence of highly productive or exploited grounds. An increase in the hotspot value of a given area may indicate intensified fishing effort, as observed in the southwest area of Julia Percy Island and the center of Julia Bank. Hence, it would be the responsibility of managers to decide whether this increase in effort is sustainable within the context of additional data such as stock density and abundance and, if not, whether downward revision in total allowable catch should be implemented for the Island. Indeed catch targets on the three subzones considered in this study were set to zero for the 2013–14 fishing season, and only a small catch target was set for Julia Percy Island during 2014–15. Conversely, a diminishing CPUE hotspot might indicate that an area is becoming less productive, or there has been translocation of effort to a different location. The patterns observed and increased intensity of effort at these locations may also be associated with management changes such as the closure of AVG affected reefs during limiting the total available reef estate for fishing. Displacement of effort as a consequence of disease was the primary reason for focusing effort on reefs on Julia Bank and in Discovery Bay that were seldom fished during the years prior to AVG. Furthermore, stronger spatial clusters and temporal shifts in CPUE were observed between 2010 and 2011 especially at Julia Bank subzone which is likely to be due to the concentration of divers’ effort to meet total allowable catch. The greater depth and patchy distribution away from the central Julia Reef area also meant that more exploratory searching was required to locate dense aggregations that yielded acceptably high CPUE.

Interpreting patterns in fishing pressure nonetheless warrants a cautious approach, because observed patterns might be dependent on the extent to which CPUE is associated with habitat structure and the occurrence of target species [55, 56]. In this study, GAMM indicated that CPUE was associated with reef complexes in shallow waters that exhibit high seafloor complexity data. Observed coincidences between CPUE and seafloor structure is further supported by a previous study that showed key blacklip abalone fishing grounds were associated with topographically complex reefs [57]. In addition, it has been well documented that depth and reef complexity represent fundamental characteristics of benthic marine ecosystems that affect a multitude of processes, including species richness and diversity [14, 58–61]. Consequently, along with the depth and the cost of operations, reef complexity is likely to influence fishing behavior considering that fishers do not knowingly expend search effort in areas where the target species is likely to be sparse or absent. In addition, CPUE mainly occurred between depths of 5 to 20 m. In some locations, major reef complexes also occur in areas deeper than 20 m, yet these areas were not completely targeted with 100% coverage. Rather divers appeared to be targeting specific areas that show these areas are perhaps more productive. For example, we observed that only ~30% and 60% of the reef areas at Julia Bank and Discovery Bay were respectively targeted by fishermen over four years. This may primarily be because of the increased risks associated with hyperbaric exposure at these depths, but possibly also due to perceived lower abalone biomass in these areas that would militate against achieving acceptable catch rates. More research should be undertaken to help better address the question of whether particular subzones that are subject to intense fishing effort support persistent clusters of abalone stocks. Extension of current knowledge about fishing-induced effects on abalone patches should enable commercial abalone divers to make better informed decisions about how intensively they should fish specific subzones to ensure that sufficient abalone resources persist for the fishery to be sustained into the future.

## Conclusions

The analyses of fishing effort based on GPS tracking using hotspot analyses facilitated the determination of CPUE intensity over time, and provided an opportunity to analyze the spatial dynamics of the fishery at a localized reef scale. Abalone CPUE was unequally distributed across the grounds, with observed trends appearing to be primarily concentrated in relatively fine-scale areas with a history of high effort. In addition, the bathymetric data further illustrated that CPUE patterns closely coincided with seafloor type (i.e. shallow reef complexes). The effective visualization and communication of these data to stakeholders (the commercial diving organizations), could potentially provide a unique opportunity to establish a useful feedback mechanism for integrating fisher knowledge into the fishery management system.
